# Impact of delayed hyperenhancement obtained by non-contrast computed tomography following coronary angiography in patients undergoing extracorporeal cardiopulmonary resuscitation

**DOI:** 10.1016/j.resplu.2020.100028

**Published:** 2020-09-28

**Authors:** Tomomi Watanabe, Toshihiko Akasaka, Naoko Sasaki, Natsuko Mukai-Yatagai, Kazuhiro Yamamoto

**Affiliations:** Department of Cardiovascular Medicine and Endocrinology and Metabolism, Faculty of Medicine, Tottori University, 86 Nishi-cho, Yonago, Tottori, 683-8503, Japan

**Keywords:** Extracorporeal cardiopulmonary resuscitation, Computed tomography, Delayed hyperenhancement

## Abstract

**Background:**

Extracorporeal cardiopulmonary resuscitation (ECPR) has the potential to improve outcomes in patients with refractory cardiac arrest. However, the outcome is difficult to predict on admission. Recent reports have described early evaluation of myocardial damage in patients with acute myocardial infarction by detecting delayed enhancement in non-contrast computed tomography (CT) following coronary angiography (CAG). We investigated the impact of delayed hyperenhancement obtained by non-contrast CT following CAG in patients with ischaemic and non-ischaemic cardiovascular diseases who underwent ECPR for refractory cardiac arrest.

**Methods:**

Forty-two patients who underwent ECPR, CAG, and postprocedural CT for refractory cardiac arrest in our institute were retrospectively enrolled. Two blinded readers independently and semi-quantitatively judged whether hyperenhancement was present or absent in non-contrast axial CT images following CAG. We evaluated the relationship between in-hospital death and delayed hyperenhancement.

**Results:**

The identification of delayed hyperenhancement was highly consistent between the two readers (kappa ​= ​0.71). The survival rate was 21.4% in this cohort. The only significant difference between the survival group and in-hospital death group was the presence of delayed hyperenhancement, which was detected only in the in-hospital death group (p ​= ​0.03). The prevalence of cardiac death was higher in patients with than without delayed hyperenhancement. Delayed hyperenhancement was observed even in areas perfused by non-obstructive coronary arteries.

**Conclusions:**

Delayed hyperenhancement of the left ventricular wall on non-contrast CT imaging following CAG might help to predict in-hospital death in patients undergoing ECPR for refractory cardiac arrest.

## Introduction

Extracorporeal cardiopulmonary resuscitation (ECPR) has the potential to improve survival and neurological outcomes compared to conventional cardiopulmonary resuscitation (CPR) in patients with refractory cardiac arrest. Although some studies have reported the advantages of ECPR in survival and neurologic outcomes in specific patient group,^1^, ^2^, ^3^ the use of ECPR still has only inconclusive evidence for out-of-hospital cardiac arrest (OHCA) and in-hospital cardiac arrest (IHCA).^4^ Furthermore, the survival rate of patients undergoing ECPR is only 20%–30%^5^ and varies among institutes and individual patients.^6^^,^^7^ Although prognostic factors for these patients have been reported,^8^^,^^9^ the outcome is difficult to predict at the initial presentation.

Early evaluation of myocardial viability in patients with acute myocardial infarction by non-contrast computed tomography (CT) following coronary angiography (CAG) has recently been reported.^10^ Myocardial delayed contrast enhancement as shown by this imaging method was related to a higher risk of cardiac events.^11^ CAG is frequently conducted in patients undergoing ECPR because coronary artery disease is a common cause of refractory cardiac arrest.^12^ However, the usefulness of delayed hyperenhancement of the left ventricular wall in non-contrast CT after CAG is unclear in such patients.

We investigated the impact of delayed hyperenhancement obtained by non-contrast CT following CAG in patients with ischaemic and non-ischaemic cardiovascular diseases who underwent ECPR for refractory cardiac arrest.

## Methods

### Study population and design

The Tottori University institutional review board approved this retrospective study. Research information regarding this study was released to the public in accordance with the guiding principles for epidemiological studies established by the Ministry of Health, Labour and Welfare, Japan. We were allowed to collect and analyse the data without obtaining written informed consent from each patient.

We investigated 82 patients who underwent ECPR for refractory cardiac arrest in Tottori University Hospital from April 2009 to February 2020. Forty-two of the 82 patients underwent non-contrast CT following diagnostic CAG or percutaneous coronary intervention with ECPR and were enrolled in this study. In our institution ECPR is indicated for OHCA patients who meet all of the followings 1) age <75 years old, 2) initial shockable rhythm, 3) starting CPR within 15 ​min from onset, 4) ECPR installation achievable within 60 ​min from onset. However, final decision was left to the primary physicians. In the case of IHCA, witness or early onset refractory cardiopulmonary arrest was indicated for ECPR except for contraindications such as haemorrhage and existence of do-not-resuscitate orders.

If the ECMO flow was stable, patients basically underwent CAG. Exceptionally, in cases with high suspicion of non-cardiac cause, CAG was omitted. Although whole body and brain CT were routinely performed, postprocedural CT was omitted when the patient was unstable to undergo whole body CT mainly due to uncontrolled bleeding or when the cardiac cause was clear, the duration of CPR was very brief, and it was judged by the attending physician as unnecessary to search for the cause of arrest or the iatrogenic injury on CT.

To explore the factors associated with in-hospital death, the patients were divided into a survival group and in-hospital death group, and the following indices were compared: clinical characteristics, clinical time courses, blood examination results, CT evaluation findings, and procedures. The clinical time courses included the onset to extracorporeal membrane oxygenation (ECMO) time, procedure time and cath lab to CT time. The procedure time was defined as the duration of time in the catheter laboratory. Echocardiography was routinely performed after CT scan at patient’s bedside and left ventricular ejection fraction (LVEF) was calculated as previously described.^13^ Blood samples were obtained upon arrival at the emergency room or catheter laboratory.

Successful weaning of ECMO was defined as survival for >30 days after ECMO weaning without a subsequent need for mechanical support.^14^ Major bleeding was defined as Bleeding Academic Research Consortium type 3 or 5 bleeding.^15^ The cause of death was classified based on a previous report,^16^ and we added “bleeding during ECMO” which indicated the unmanageable bleeding with critically decreasing ECMO flow unresponsive to volume load including transfusion. Cognitive function was assessed by the Cerebral Performance Category (CPC) at discharge.

### ECPR management

In all ECPR procedures, veno-arterial (VA)-ECMO was introduced in the catheter laboratory using a percutaneous technique under conventional CPR with a mechanical chest compression–decompression device (Lund University Cardiopulmonary Assist System: LUCAS™). The pump flow was initially set at >3.0 ​L/min, and 5000 units of heparin was administrated. All patients underwent CAG and, if necessary, coronary intervention. To assess bleeding complications and hypoxic brain injury, whole-body CT scanning was conducted on VA-ECMO after cardiac catheterisation. Targeted temperature management was performed in all patients. Basically, normothermia was adopted in almost all patients; hypothermia (core temperature maintained at 35 ​°C for 24 ​h followed by rewarming at 0.1 ​°C per hour to a maintenance temperature of 36 ​°C) was selected in patients who were haemodynamically stable without major bleeding. After rewarming periods, once sedation was stopped and we assessed the degree of the hypoxic brain injury using restudy of brain CT and electroencephalography, if necessary. However, even if severe anoxic brain injury was suspected at 72 ​h after cardiac arrest, active withholding of the care or active withdrawing of ECPR was not chosen in any patients with OHCA at least for a few weeks in our institution.

### CT scanning protocol

Following cardiac catheterisation, non-electrocardiogram (ECG)-gated plain CT was performed using a 64-row multidetector CT scanner (Aquilion CX; Canon (Toshiba) Medial Systems, Tochigi, Japan) as previously described.^17^

### CT image analysis

**Fig. 1:**
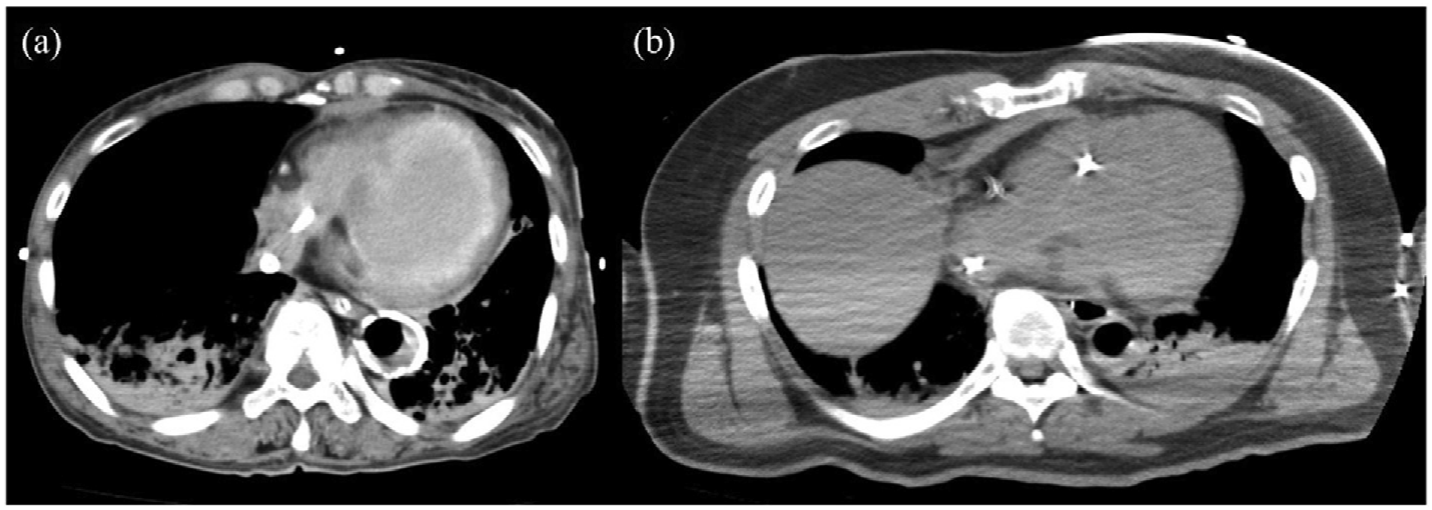
Representative images of the (a) presence and (b) absence of delayed hyperenhancement. (a) Hyperenhancement is distributed in a broad area on the endocardium of the left ventricular wall. (b) No staining is detected in the left ventricular wall.

Two cardiologists who were blinded to the diagnosis acted as readers and independently analysed the non-ECG-gated CT axial images. The presence of delayed hyperenhancement was defined as apparent high attenuation in the myocardium, excluding artefacts such as the VA-ECMO cannula. In judging the presence of delayed hyperenhancement, the left ventricular wall was divided into three segments based on the territories of the coronary arteries: anterior, lateral, and inferior.^18^ The probability of delayed hyperenhancement was evaluated using a 5-point confidence scale (1, definitely absent; 2, probably absent; 3, equivocal; 4, probably present; and 5, definitely present).^19^ Patients with images ranked as definitely present or probably present were classified as having delayed hyperenhancement. Representative cases are shown in Fig. 1.

### Statistical analysis

Normally distributed continuous variables are reported as mean ​± ​standard deviation, and non-normally distributed continuous variables are reported as median (25th percentile, 75th percentile). Categorical variables are reported as percentages. Student’s t-test was used to compare normally distributed continuous variables, and the Mann–Whitney *U* test was used to compare non-normally distributed continuous variables. Pearson’s chi square test was used to compare categorical variables between the two groups. Kappa statistics were used to determine the reproducibility of evaluations between the two readers. Statistical significance was defined as p ​< ​0.05. IBM SPSS version 25 (IBM Corp., Armonk, NY, USA) was used for all statistical analyses.

## Results

### Interobserver reproducibility of delayed hyperenhancement

The identification of delayed enhancement was highly consistent between the two readers (kappa ​= ​0.71). The kappa value for the anterior, lateral, and inferior location was 0.76, 0.67, and 0.59, respectively. Discrepancies between the readers were resolved by discussion after both reading sessions were completed.

### Patient characteristics

The survival rate was 21.4% in this cohort (19.2% for OHCA and 25.0% for IHCA). There were no significant differences between the survival group and in-hospital death group in terms of patient characteristics, clinical time courses, initial blood parameters, or procedure characteristics except for the CT evaluation (Table 1). Only the prevalence of delayed hyperenhancement was significantly different between the two groups. Delayed hyperenhancement was detected only in the in-hospital death group.

**Table 1:** Patient characteristics and clinical time courses.

	All patients (n ​= ​42)	Survive (n ​= ​9)	In-hospital death (n ​= ​33)
*Clinical characteristics*			
Age, years	66.5 (55.7, 73.3)	67.0 (43.5, 74.0)	66.0 (56.5, 73.5)
Males, n (%)	38 (90)	8 (89)	30 (91)
OHCA, n (%)	26 (62)	5 (56)	21 (64)
Ischemic causes, n (%)	30 (71)	5 (56)	25 (76)
Bystander CPR, n (%)	26 (62)	3 (33)	23 (70)
Initial shockable rhythm, n (%)	24 (57)	6 (67)	18 (55)
Prior myocardial infarction, n (%)	5 (12)	1 (11)	4 (12)
LVEF, %	20.0 (10.0, 30.0)	20.0 (10.0, 42.5)	20.0 (10.0, 30.0)
Left main disease, n (%)	5 (12)	1 (11)	4 (12)
Multi-vessel disease, n (%)	22 (52)	3 (33)	19 (58)
*Clinical time courses*			
Onset to ECMO time, min	60.0 (47.0, 80.0)	57.0 (51.25, 74.8)	62.0 (46.0, 84.5)
Procedure time, min	120.0 (78.8, 167.3)	110.0 (87.5, 167.5)	120.0 (77.5, 167.4)
*Blood samples*			
pH	7.12 ​± ​0.2	7.2 ​± ​0.2	7.1 ​± ​0.2
Lactate, mmol/L	9.7 (7.2, 12.9)	9.3 (6.8, 14.1)	9.7 (7.2, 12.8)
Hemoglobin, g/dL	12.5 ​± ​2.4	12.5 ​± ​1.7	12.6 ​± ​2.6
Platelet count, ​× ​10^4^/μL	15.7 (10.2, 14.0)	13.2 (8.6, 17.5)	16.5 (11.4, 23.4)1
D-dimer, μg/mL	5.6 (2.2, 26.3)	10.7 (0.8, 26.9)	5.6 (2.3, 25.5)
Creatinine, mg/dL	1.1 (1.0, 1.4)	1.0 (0.9, 1.6)	1.2 (1.0, 1.4)
initial CK, IU/L	174.5 (112.8, 479.5)	147.0 (89.0, 306.5)	176.0 (125.5, 734.5)
initial CK-MB, IU/L	84.1 (23.5, 97.0)	32.0 (13.5, 97.5)	58.5 (26.3, 98.0)
peak CK, IU/L	5084.5 (2223.5, 10571.8)	7135.0 (3261.5, 16145.0)	4258.0 (2056.0, 10328.5)
peak CK-MB, IU/L	338.5 (171.8, 851.3)	453.0 (130.5, 744.5)	325.0 (179.5, 946.0)
*CT evaluation*			
Delayed hyperenhancement, n (%)	12 (29)	0 (0)	12 (36)
*Procedures*			
Hypothermia, n (%)	10 (24)	2 (22)	8 (24)
IABP, n (%)	26 (62)	7 (78)	19 (58)
PCI, n (%)	22 (52)	4 (44)	18 (55)
Transfusion at 1st day, units	5.0 (0, 8.5)	2.0 (0.0, 7.0)	6.0 (0.0, 10.0)
*Outcomes*			
Successful weaning from ECMO, n (%)	14 (33)	9 (100)	5 (15)
Major bleeding, n (%)	22 (52)	3 (33)	19 (58)
Cerebral Performance Category	5.0 (5.0, 5.0)	3.0 (1.0, 3.75)	5.0 (5.0, 5.0)
Cause of death			
Bleeding during ECMO, n (%)	13 (31)		13 (39)
Multi organ failure, n (%)	3 (7)		3 (9)
Cardiac death, n (%)	12 (29)		12 (36)
Other/unknown, n (%)	5 (12)		5 (15)

Normally distributed continuous variables are reported as mean ​± ​standard deviation, and non-normally distributed continuous variables are reported as median (25th percentile, 75th percentile).

OHCA: out of hospital cardiac arrest, LVEF: left ventricular ejection fraction, ECMO: extracorporeal membrane oxygenation, CPR: cardiopulmonary resuscitation, CK: creatine kinase, CK-MB: creatine kinase isoenzyme MB, IABP: intra aortic balloon pumping, PCI: percutaneous coronary intervention.

To identify factors related to delayed hyperenhancement, we compared clinical characteristics, including the amount of contrast medium and cath lab to CT time, between 12 patients with delayed hyperenhancement and the remaining 30 patients without delayed hyperenhancement (Table 2). There was no significant difference in clinical characteristics including LVEF, cath lab to CT time and contrast medium between 2 groups. Cardiac death was more frequent in patients with delayed hyperenhancement. Because all patients with delayed hyperenhancement dead in hospital, the impact of the delayed hyperenhancement on cognitive function could not be evaluated.

**Table 2:** Characteristics in patients of delayed hyperenhancement.

	Delayed hyperenhancement negative (n ​= ​30)	Delayed hyperenhancement positive (n ​= ​12)	P value
*Clinical characteristics*			
Age, years	66.0 (54.8, 73.3))	67.5(59.3, 74.5)	0.65
Ischemic causes, n (%)	20 (67)	10 (83)	0.19
Initial shockable rhythm, n (%)	19 (63)	5 (42)	0.17
Prior myocardial infarction, n (%)	3 (10)	2 (17)	0.45
LVEF, %	20.0 (10.0, 30.0)	17.0 (10.0, 25.0)	0.54
Left main disease, n (%)	3 (10)	2 (17)	0.45
Multi-vessel disease, n (%)	15 (50)	7 (58)	0.44
*Clinical time courses*			
Onset to ECMO time, min	62.0 (51.5, 84.5)	57.0 (35.8, 76.0)	0.31
Procedure time, min	127.7 ​± ​44.0	109.5 ​± ​44.2	0.24
Cath lab to CT time, min	10.0 (8.0, 16.0)	7.0 (3.8, 21.8)	0.37
*Others*			
Contrast medium, mL	100.5 ​± ​54.0	139.5 ​± ​68.6	0.08
Peak CK-MB, IU/L	317.0 (186.5, 747.3)	641 (129.5, 1463.3)	0.39
*Procedure*			
Hypothermia, n (%)	7 (23)	3 (25)	0.60
PCI, n (%)	14 (47)	8 (67)	0.20
*Outcomes*			
Successful weaning from ECMO, n (%)	10 (33)	1 (8)	0.10
Cerebral Performance Category	5.0 (4.0, 5.0)	5.0 (5.0, 5.0)	0.18
30 days mortality, n (%)	20 (67)	10 (83)	0.25
Cardiac death, n (%)	5 (17)	7 (58)	0.01

Normally distributed continuous variables are reported as mean ​± ​standard deviation, and non-normally distributed continuous variables are reported as median (25th percentile, 75th percentile).

LVEF: left ventricular ejection fraction, ECMO: extracorporeal membrane oxygenation, CK-MB: creatine kinase isoenzyme MB, PCI: percutaneous coronary intervention.

### Details of patients with delayed hyperenhancement

The details of the 12 patients with delayed hyperenhancement are shown in Table 3. The major cause of death was cardiac death (n ​= ​7). Delayed hyperenhancement was observed even in territories perfused by non-obstructive coronary arteries in six patients.

**Table 3:** Details of the cases with delayed hyperenhancement.

	Initial rhythm	Diagnosis	Onset to ECMO, min	Location of delayed hyperenhancement	Major bleeding	Peak CK, IU/L	Weaning from ECMO	Cause of death
Case 1	VF	idiopathic	62	lat, inf	yes	9549	no	bleeding
Case 2	PEA	AMI	77	ant, lat, inf	yes	2028	no	cardiac death
Case 6	VF	Idiopathic	84	ant, lat	no	148	no	Unknown
Case 13	PEA	AMI	47	ant, lat	no	27182	LVAD	cardiac death
Case 14	PEA	AMI	14	ant, lat	no	22200	no	cardiac death
Case 17	Asys	AMI	73	ant, lat	yes	144	no	Bleeding
Case 22	PEA	AMI	10	ant	yes	264	no	Bleeding
Case 25	VF	AMI	77	lat, inf	yes	5718	no	Bleeding
Case 26	PEA	AMI	54	ant, lat	yes	17150	yes	cardiac death
Case 28	PEA	AMI	32	ant, lat, inf	no	3956	no	cardiac death
Case 31	VF	AMI	60	ant	no	24894	no	cardiac death
Case 34	VF	AMI	153	ant, inf	no	2270	no	cardiac death

ECMO: extracorporeal membrane oxygenation, PCI: percutaneous coronary intervention, CK: creatine kinase, VF: ventricular fibrillation, PEA: pulseless electrical activity, Asys: Asystole, AMI: acute myocardial infarction, RCA: right coronary artery, LMT: left main trunk, LAD: left anterior descending, lat: lateral, inf: inferior, ant: anterior, LVAD: left ventricular assist device.

## Discussion

Among patients undergoing ECPR for refractory cardiac arrest, delayed hyperenhancement on non-contrast CT following CAG was found even in non-culprit sites and in patients without coronary artery diseases. Delayed hyperenhancement was associated with in-hospital death.

It is important to predict the prognosis of patients who undergo ECPR for refractory cardiac arrest in terms of considering the need for escalation of intensive care. During treatment with ECPR, examinations are limited to avoid equipment-related complications such as accidental cannula disconnection during patient transfer. A whole-body CT scan is a reasonable examination in patients undergoing ECPR to detect the cause of arrest or iatrogenic trauma.^20^ A contrast-enhanced CT image of the left ventricular wall in patients with acute myocardial infarction provides two findings in different phases: early defects and late enhancement.^21^ Our previous work and other studies have revealed the clinical impact of both early defects^17^^,^^22^^,^^23^ and late enhancement^10^^,^^11^^,^^24^ in patients with acute coronary syndrome. Sugiyama et al.^25^ recently reported that an early defect in the left ventricular wall on contrast-enhanced CT images in patients with out-of-hospital cardiac arrest undergoing ECPR was associated with poor outcomes; however, the clinical implication of late enhancement in the left ventricular wall and the usefulness of non-contrast CT images following CAG in patients undergoing ECPR remain unclear. The current study expanded the previous findings by demonstrating that delayed hyperenhancement in the left ventricular wall on non-contrast CT images following CAG is associated with a poor prognosis in patients with ischaemic and non-ischaemic cardiovascular diseases who undergo ECPR for refractory cardiac arrest. Furthermore, delayed hyperenhancement was detected even in regions perfused by non-culprit or non-obstructive coronary arteries, and the relationship between delayed hyperenhancement and the prognosis could be applied to patients without coronary artery disease. CT imaging after CAG may provide further information without the need for additional contrast agent or procedures to predict the prognosis of patients treated with ECPR.

Our recent study demonstrated excellent agreement of myocardial delayed enhancement on CT with that on cardiac magnetic resonance imaging.^19^ Habis et al.^10^ reported that late hyperenhancement of the left ventricular wall on non-contrast CT imaging soon after CAG in patients with acute myocardial infarction indicates a lack of myocardial viability. Therefore, delayed hyperenhancement in regions perfused by non-culprit or non-obstructive coronary arteries may represent myocardial injury at least partly caused by a mismatch between myocardial oxygen demand and supply, which explains the association between the delayed hyperenhancement and the poor prognosis in the present study. More intensive treatment to reduce myocardial oxygen demand through the unloading of the left ventricle may be required for patients with delayed hyperenhancement in the region perfused by non-obstructive coronary arteries. However, this simple and quick assessment with non-contrast CT after CAG may help in the decision-making regarding transition to palliative treatment in some patients.

The semi-quantitative evaluation of delayed hyperenhancement showed good reproducibility between the two readers in this study. Notably, the kappa value of the inferior region was slightly lower than that of the other regions, which may be partly explained by the fact that the axial plane was used for the evaluation.

The present study has several limitations. The number of patients was small, we analysed in- and out-of-hospital cardiac arrest together, both patients with ischaemic and non-ischaemic cardiovascular diseases were included, and the study was retrospective in nature. The data contained large variations, especially the time courses. Thus, some important factors, such as the door-to-balloon time, were not analysed. Furthermore, it is still unknown whether delayed hyperenhancement has the same significance in all post-cardiac arrest patients, Because both of CAG and subsequent CT scan are rarely performed in post arrest patients without ECPR in our institute, we don’t have enough data to discuss the clinical significance of delayed hyperenhancement in all arrest patients. Further investigations are needed to answer this interesting issue. A quantitative assessment using CT values may be more suitable. CT images with high quality are required for the quantitative assessment, however, could not be obtained in the emergent situation of the present study due to motion and mechanical artifact. Likewise, the patterns and extents of delayed hyperenhancement were not analysed because of the poor imaging quality.

## Conclusions

Delayed hyperenhancement of the left ventricular wall obtained by non-contrast CT following CAG could be evaluated by a simple semi-quantitative method with acceptable reproducibility. It was associated with in-hospital death in patients undergoing ECPR for refractory cardiac arrest, and it might be helpful in predicting the prognosis and deciding the treatment strategy for such patients.

## Declaration of competing interest

The authors declare that they have no known competing financial interests or personal relationships that could have appeared to influence the work reported in this paper.
